# Acute Stress and Gender Effects in Sensory Gating of the Auditory Evoked Potential in Healthy Subjects

**DOI:** 10.1155/2021/8529613

**Published:** 2021-03-12

**Authors:** Zengyou Xin, Simeng Gu, Wei Wang, Yi Lei, Hong Li

**Affiliations:** ^1^School of Education Science, Minnan Normal University, Zhangzhou, Fujian 363000, China; ^2^Brain and Cognitive Neuroscience Research Center, Liaoning Normal University, Dalian, Liaoning 116029, China; ^3^Institute of Brain and Psychological Sciences, Sichuan Normal University, Chengdu, Sichuan 610060, China; ^4^Department Medical Psychology, Jiangsu University Medical School, Zhenjiang, Jiangsu 210023, China; ^5^Department of Neurosurgery, University of Rochester Medical Center, Rochester, New York 14643, USA; ^6^Center for Language and Brain, Shenzhen Institute of Neuroscience, Shenzhen Key Laboratory of Affective and Social Cognitive Science, School of Psychology, Shenzhen University, Shenzhen, 518060 Guangdong, China

## Abstract

Sensory gating is a neurophysiological measure of inhibition that is characterized by a reduction in the P_50_, N_100_, and P_200_ event-related potentials to a repeated identical stimulus. It was proposed that abnormal sensory gating is involved in the neural pathological basis of some severe mental disorders. Since then, the prevailing application of sensory gating measures has been in the study of neuropathology associated with schizophrenia and so on. However, sensory gating is not only trait-like but can be also state-like, and measures of sensory gating seemed to be affected by several factors in healthy subjects. The objective of this work was to clarify the roles of acute stress and gender in sensory gating. Data showed acute stress impaired inhibition of P_50_ to the second click in the paired-click paradigm without effects on sensory registration leading to worse P_50_ sensory gating and disrupted attention allocation reflected by attenuated P_200_ responses than control condition, without gender effects. As for N_100_ and P_200_ gating, women showed slightly better than men without effects of acute stress. Data also showed slightly larger N_100_ amplitudes across clicks and significant larger P_200_ amplitude to the first click for women, suggesting that women might be more alert than men.

## 1. Introduction

Acute stress is a stereotypical and multimodal response to a present or imminent challenge overcharging an organism [1] and causes the release of many stress hormones and neuromodulators (e.g., [2]), which can change cellular properties of large-scale neuronal populations throughout the brain [3]. The common stress induction paradigms in laboratory include cold water pressing (also called cold pressor stress (CPS); e.g., [4]) and the Trier Social Stress Test (TSST; [5]). Sensory gating is the ability of the central nervous system to filter incoming stimuli and protect a person from being flooded with irrelevant information (e.g., [6]). It is typically measured by using a paired-click paradigm. In such a paradigm, the event-related potential P_50_ is measured during the presentation of two identical clicks with an interstimulus interval (ISI) of 500 ms and a typical interpair interval (IPI) no less than 8 s [7]. The attenuation of the P_50_ amplitude to the second click (*S*_2_), relative to that of the first click (*S*_1_), is the operational definition of gating inhibition (i.e., gating or sensory gating; [8, 9]). The difference and the ratio between these P_50_ responses are the means to quantitatively assess gating mechanisms (e.g., [9–11]).

Freedman and colleagues initially demonstrated that schizophrenic patients and their family members fail to exhibit typical P_50_ response inhibition to *S*_2_ during a paired-click paradigm (e.g., [8, 12]). Since then, sensory gating has been one of the foci of psychopathological researches on schizophrenia. Because of the highly replicable results across studies on schizophrenia (e.g., [13–16]) and the findings that filtering deficits also occur in unaffected family members [17, 18], P_50_ filtering has been investigated as a candidate potential endophenotype for schizophrenia. Additionally, in psychopathological studies, there also have been concerns on N_100_ and P_200_ sensory gating, which were assessed using event-related potential N_100_ and P_200_, respectively. However, as for normative data regarding the characteristics of sensory gating, up till now, relatively limited studies using healthy volunteers have targeted gender and acute stress, respectively, and have reported inconsistent outcomes (see Table 1).

According to previous studies focusing on gender difference, three studies supported less P_50_ sensory gating for women [10, 19, 23], and six of the limited documents support no gender difference in P_50_ sensory gating. The similarly inconsistent findings on the P_50_ amplitudes for *S*_1_ or *S*_2_ were reported between genders. As for the influences of acute stress, although adverse P_50_ sensory gating effects were reported in almost all studies (except for Woods et al. [11]), there were inconsistent findings on the P_50_ response to the two clicks after acute stress intervention. As for auditory evoked potential components other than P_50_, as shown in Table 1, the results of N_100_ and P_200_ seemed even more inconsistent.

To date, except for a single study, no attempt has been made to investigate the gender difference in the effects of acute stress on sensory gating. During an oral mental arithmetic stressor task, sensory gating ratios were measured to the paired-click paradigm, and women showed disrupted P_50_ and N_100_ gating whereas men exhibited only disrupted N_100_ gating [12]. Although White et al. reported valuable information, several limitations have to be noted. The first is the protocol used to induce acute stress. According to White et al. [12], P_50_ data were recorded while mental arithmetic was performing, in which the experimental effect was inevitably contaminated by the concurrent working memory task; however, P_50_ sensory gating was not a complete automatic and preattentive process; it involved top-down modulations and could be influenced by attention manipulation [16, 31, 32]. Furthermore, the muscle artifacts of oral mental arithmetic cannot be ignored. The second is the different interpair intervals (10-14 s for some subjects, while 7-10 s for others), which may deeply affect the P_50_ response (e.g., [7]), because the sensory gating data were derived from two different studies [27, 28]. The third is numbers of epochs averaged (80 trials for data from White and Yee [27], 60 trials from Yee and White [28]), and the fourth is limited sample size (13 women and 16 men), in addition to some differences in experimental procedures.

Thus, the main objective of this study was to further investigate possible gender differences in the sensory gating after exposure to stressful treatment or control condition. It has been reported that the fluctuation of sex steroid hormone during menstrual cycles affects the performance of working memory [33], proponent response inhibition [34], arousal state [35], and fear conditioning and inhibition [36] in healthy women. Thus, only women during their midluteal phase were included to control the potential effects of menstrual cycle. A body of literature suggests the major neural sources of P_50_ suppression involves the hippocampus and prefrontal cortex (e.g., [9, 37, 38]), where stress hormone receptors are abundant and stress exerts effects on cognitive processes (e.g., [3, 39, 40]). Therefore, sensory gating under acute stress would therefore be expected to be impaired. Additionally, it has been supposed that two processes contribute to the gating deficit, i.e., a reduced sensory registration (*S*_1_ amplitude reduction) and a reduced ability to inhibit the repeated auditory stimulation (lack of reduction of *S*_2_ amplitude; e.g., [41–43]). Sensory registration and suppression may be differentially affected by acute stress and may show gender difference meanwhile. Thus, another aim of this study is to determine which one plays a big role in the gender and acute stress effects. Given that fewer studies using paired-click paradigm focused on N_100_ and P_200_ gating, an exploratory investigation was also made in the current study.

## 2. Method

### 2.1. Participants

Forty-three healthy university students (25 males, 18 females) were included in the study. One female subject dropped out, data of a male subject was incomplete because of technical failure, and another female was excluded because of failure to obtain reliable P_50_ response in CPS experimental session. Thus, data were from 24 men (ranged in age from 20 to 23 years, mean = 21.3, SD = 0.86) and 16 women (for N_100_ and P_200_ data; ranged from 19 to 23; mean = 20.76, SD = 0.97). Statistical results of 16 women other than P_50_ data would not be present in the paper because there was no significant difference from 17-woman sample that were included in the analyses. Only women during their midluteal phase (with regular menstrual cycles, days 16 to 24) were included to control possible gender and ovarian cycle effects on adrenocortical reactivity [44, 45]. Participants were asked to refrain from caffeine, alcohol, tea, and smoking within 6 h before the experimental sessions. The volunteers were recruited by announcements and received financial compensation. The study was approved by the Ethics Committee of the Minnan Normal University. All participants were naive to the purpose of the study and gave their written informed consent prior to their inclusion in the study.

### 2.2. Procedure

After a participant's arrival, he or she was allowed to rest briefly, and then, preexperimental saliva sample (to measure cortisol level) was taken, and systolic blood pressure (SBP), diastolic blood pressure (DBP), and heart rate (HR) were recorded at the same time to evaluate participant's physical baseline. Then, participants filled out the Positive and Negative Affect Schedule (PANAS; [46]), Beck Depression Inventory-Second Edition (BDI-II; [47]), and Anxiety Inventory [48]. Data of Trait Anxiety Inventory was collected during their first experimental session. Then, participants were exposed to either the CPS treatment or the warm water (control) treatment (adapted from) [4]. Immediately after treatment, all subjects had a rest, and then SBP, DBP, and HR were measured at about 4 min after CPS or control procedures. Then, subjects were engaged in the experimental task, and meanwhile, EEG data was collected. Further, saliva samples were taken immediately after the task (about 15 min after treatment). The method of salivary cortisol measurement was described in Yang et al. [49]. All salivary samples were stored at -40°C, and analyses were completed within about one month.

This experiment was conducted by adopting a within-subjects design, in which CPS and control procedures were, respectively, applied by an interval of at least 24 h, and treatment order was counterbalanced. Subjects were instructed to submerge their hands and wrists in cold water (6° to 9°C) for 5 min for CPS session while in warm water (35° to 38°C) during control session (adapted from [4]). To avoid any influence of the circadian profiles of adrenocortical reactivity and cognitive ability, CPS or control procedures were conducted in the same time period of the experiment day and the other experimental procedures were the very same.

### 2.3. Stimuli and EEG Recording and Analysis

#### 2.3.1. Auditory Stimulation

About 5 min after treatment, paired clicks of 2000 Hz, 95 dB SPL tones, and 4 ms in duration were delivered via headphones with 50 dB SPL background white noise. The sound intensity was measured at the subject's ear by a sound meter. All 60 paired clicks were separated by a 500 ms interval, and interpair interval was random ranged from 7.5 to 10 s in order to allow brain activity to return to baseline (e.g., [6, 7]).

#### 2.3.2. EEG Recording

Participants were seated in a comfortable chair in an electromagnetically shielded room, wearing headphones, and instructed to sit comfortably and still, close their eyes, relax, and listen to clicks. All subjects were monitored for signs of drowsiness by visual observation and EEG monitoring because P_50_ component is sleep-state dependent (e.g., [11]). Brain electrical activity was recorded at F_z_, C_z_, P_z_, F_3_, F_4_, C_3_, C_4_, O_1_, and O_2_ sites using Ag/AgCl electrodes mounted on an elastic cap (Brain Product, München, Germany), with references on FC_z_, and a ground electrode on the medial frontal aspect. Vertical electrooculograms (EOGs) were recorded supra- and infraorbitally at the left eye. The horizontal EOG was recorded from the left versus right orbital rim. The EEG and EOG were amplified using a 0.05 to 100 Hz bandpass and were continuously digitized at 1000 Hz/channel during online recording. All interelectrode impedances were maintained below 5 k*Ω*.

#### 2.3.3. EEG Analysis

Offline, the data were referenced to the average of the left and right mastoids, digitally filtered at 10–50 Hz for P_50_ and 1–30 Hz for N_100_ and P_200_, a 50 Hz notch filter and both a roll-off of 24 dB/octave, segmented (–100 to 200 ms for P_50_; –100 to 400 ms for N_100_ and P_200_), and baseline-corrected (100 ms). Trials containing artifacts (activity in any channel exceeded 75 *μ*V) were removed from further analyses. Totally, 75–100% of the epochs (45–60 trials) were included in the N_100_ and P_200_ analyses and 77-100% (46-60 trials) for P_50_. There is no statistical difference in number of epochs of each condition.

After averaging, according to the procedures of former studies (e.g., [11, 12, 16, 27, 38]), latencies and amplitudes of the P_30_, N_40_, P_50_, N_100_, and P_200_ at C_z_ were analyzed on the basis of automatic peak detection in combination with a visual inspection according to the waveforms drawn using Excel 2007. The P_50_ component was defined as the most positive response between 45 and 90 ms poststimulus preceded by a P_30_ wave in a 15–45 ms range. The N_100_ and P_200_ component was defined as a prominent negative-positive complex (N_100_: 80–180 ms, P_200_: 120–250 ms). P_50_ amplitude was normally measured relative to the N_40_ (defined as the most negative response between P_30_ and P_50_ latencies; if no identifiable P_30_, then between 30 ms and P_50_ latency). If no identifiable N_40_ happened under any condition, all P_50_ amplitudes of this subject were measured relative to the prestimulus baseline, and this solution was also used in case of negative P_50_ gating ratios. N_100_ and P_200_ amplitudes were measured relative to the prestimulus baseline. As for components of *S*_2_, they were additionally determined by reference to the *S*_1_ component latencies (i.e., ±15 ms away from latency of *S*_1_ P_50_ for *S*_2_ P_50_, ±30 ms for *S*_2_ N_100_ and ±35 ms for *S*_2_ P_200_). When no amplitude was identifiable for *S*_1_, the subject's response was excluded from further analysis (one case for P_50_). If this was the case for *S*_2_, it was interpreted as maximum suppression and the amplitude was set to zero in accordance [50]. Gating indices were calculated as gating ratio (*S*_2_/*S*_1_ × 100) as well as gating difference (*S*_1_ − *S*_2_).

## 3. Results

### 3.1. Mood, Trait Anxiety, and Physiological Measurements

Results of trait anxiety test demonstrated no difference between males (*M* = 40.5, SD = 4.7) and females (*M* = 40.8, SD = 4.9, *p* = 0.85). To evaluate potential differences in baseline mood variables (positive and negative affect, state anxiety, and depression), mixed measure ANOVAs were conducted with treatment (CPS vs. control) as a within-subjects factor and gender as a between-subjects factor. The ANOVA showed no main effects of gender (*p* = 0.86, 0.29, 0.48, and 0.59, respectively), treatment (*p* = 0.44, 0.64, 0.24, and 0.77, respectively), and no interactions (*p* = 0.99, 0.22, 0.22, and 0.88, respectively) (Table 2). Mixed measure ANOVAs were also conducted on cardiovascular and cortisol reactivity with two within-subjects factors: treatment (CPS vs. control) and timing (baseline and after treatment) and gender as another factor to evaluate the effect of experimental manipulation. The results showed significant main effects of treatment in HR [*F* (1, 39) = 14.13, *p* = 0.001, *η*2 p = 0.27], DBP [*F* (1, 39) = 16.30, *p* < 0.001, *η*2 p = 0.30], SBP [*F* (1, 39) = 11.25, *p* = 0.002, *η*2 p = 0.22], and cortisol concentrations [*F* (1, 39) = 7.09, *p* = 0.011, *η*2 p = 0.15] and significant main effects of gender. Specifically, data showed significantly higher HR (*p* = 0.026) and cortisol concentrations (*p* = 0.024) for females, while higher DBP (*p* = 0.051) and SBP (*p* < 0.001) for males. The results also showed significant interactions of treatment × timing to DBP [*F* (1, 39) = 7.50, *p* = 0.009, *η*2 p = 0.16], SBP [*F* (1, 39) = 6.02, *p* = 0.019, *η*2 p = 0.13], and cortisol [*F* (1, 39) = 13.0, *p* = 0.001, *η*2 p = 0.25]. Further analysis showed no difference of blood pressures between baseline and after-treatment data during control session while in CPS session there were significantly higher after-treatment DBP (*p* < 0.001), SBP (*p* = 0.002), and cortisol concentrations (*p* = 0.001), relative to baseline data.

### 3.2. P_50_ Latencies, Amplitudes, and Sensory Gating

**Figure 1:**
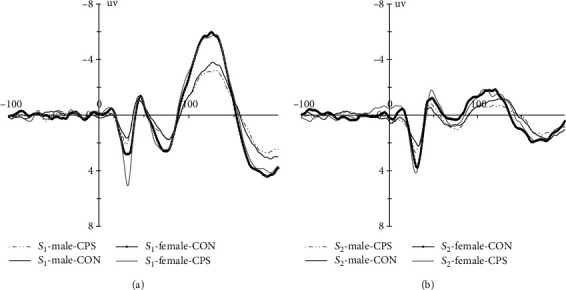
Grand averaged auditory evoked potential waves (C_z_) elicited by paired clicks ((a) *S*_1_, (b) *S*_2_) for both genders during two experimental sessions (CON: control condition; CPS: cold pressor stress treatment) (bandpass filter = 10–50 Hz).

The grand averaged auditory evoked potentials for both genders, during two experimental sessions, are presented in Figure 1, and descriptive results on P_50_ measures can be found in Table 3. P_50_ amplitudes and latencies to peak were evaluated by performing separate 2 (gender) × 2 (stimuli: *S*_1_ vs. *S*_2_) × 2 (treatment: CPS vs. control) mixed measure ANOVAs. The ANOVA showed the P_50_ latencies did not show any main effects and interactions (all *p* > 0.14). However, the P_50_ amplitudes showed a significant main effect of stimuli [*F* (1, 38) = 25.29, *p* < 0.001, *η*2 p = 0.40] and a significant interaction of stimuli × treatment [*F* (1, 38) = 17.18, *p* < 0.001, *η*2 p = 0.31], but no main effect of gender or other interactions. Further analysis showed P_50_ amplitude to *S*_2_ during CPS experimental session was significantly larger than that to *S*_2_ during control session (*p* = 0.01), while there is no difference for P_50_ amplitude to *S*_1_ between two sessions. Additionally, P_50_ amplitude to *S*_1_ was significantly larger than that to *S*_2_ for both sessions (*p* = 0.037 for CPS and *p* < 0.001 for control).

P_50_ gating ratios and gating difference were evaluated by performing 2 (gender) × 2 (treatment) mixed measure ANOVAs. The results showed no gender main effect (*p* = 0.79) and interaction (*p* = 0.85), but a significant main effect of treatment [*F* (1, 38) = 9.72, *p* = 0.003, *η*2 p = 0.20] for gating ratios. As for gating difference, the same results were obtained, which showed a better gating function after control procedures than that of CPS [*F* (1, 38) = 17.18, *p* < 0.001, *η*2 p = 0.31], without gender effect (*p* = 0.77) and interaction (*p* = 0.93).

### 3.3. N_100_ Latencies, Amplitudes, and Sensory Gating

**Figure 2:**
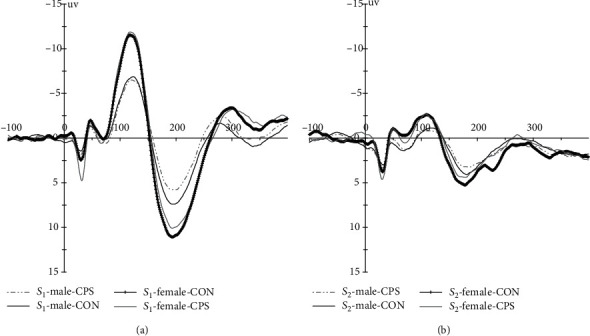
Grand averaged auditory evoked potential waveforms (C_z_) elicited by paired clicks ((a) *S*_1_, (b) *S*_2_) for both genders during two experimental sessions (bandpass filter = 1–30 Hz).

The grand averaged potentials are presented in Figure 2, and descriptive results on N_100_ are shown in Table 3. N_100_ amplitudes and latencies were evaluated by performing separate 2 (gender) ×2 (stimuli: *S*_1_ vs. *S*_2_) × 2 (treatment: CPS vs. control) mixed measure ANOVAs.

According to the ANOVA, there was a significant main effect of stimuli with longer latencies for *S*_1_ than *S*_2_ [*F* (1, 39) = 6.63, *p* = 0.014, *η*2 p = 0.15] and a borderline main effect of gender [*F* (1, 39) = 3.39, *p* = 0.073, *η*2 p = 0.08], with slightly longer latencies for males than females, but there was no main effect of treatment and no interactions on latency (all *p* > 0.67). The results also showed a significant main effect of stimuli [*F* (1, 39) = 80.92, *p* < 0.001, *η*2 p = 0.68, *S*_1_ > *S*_2_] and of gender [*F* (1, 39) = 5.14, *p* = 0.029, *η*2 p = 0.12, larger for females than males] for the mean N_100_ amplitudes and a borderline significant interaction of stimuli × gender [*F* (1, 39) = 4.04, *p* = 0.05, *η*2 p = 0.09] without treatment effect. Further analysis showed relatively larger N_100_ amplitudes in women than men for *S*_1_ (*p* = 0.028) and *S*_2_ (*p* = 0.077).

N_100_ gating ratios and gating difference were evaluated by performing 2 (gender) × 2 (treatment) mixed measure ANOVAs. The results showed no main effects of gender (*p* = 0.50) and treatment (*p* = 0.99) and no interaction (*p* = 0.51) for gating ratios. The data showed similar results for gating difference, but a borderline main effect of gender [*F* (1, 39) = 4.04, *p* = 0.05, *η*2 p = 0.09], with slightly better N_100_ gating function for females.

### 3.4. P_200_ Latencies, Amplitudes, and Sensory Gating

The grand averaged potentials are presented in Figure 2, and descriptive results of P_200_ can be found in Table 3. P_200_ amplitudes and latencies were evaluated by performing 2 (gender) × 2 (stimuli) × 2 (treatment) mixed measure ANOVAs. As for latencies, there was only a significant main effect of stimuli [*F* (1, 39) = 9.31, *p* = 0.004, *η*2 p = 0.19], with longer P_200_ latencies for *S*_1_ than *S*_2_. As for P_200_ amplitudes, the results showed there were significant main effects of treatment [*F* (1, 39) = 4.43, *p* = 0.042, *η*2 p = 0.10], stimuli [*F* (1, 39) = 37.89, *p* < 0.001, *η*2 p = 0.49], and gender [*F* (1, 39) = 6.34, *p* = 0.016, *η*2 p = 0.14] and a significant interaction of stimuli × gender [*F* (1, 39) = 4.47, *p* = 0.041, *η*2 p = 0.10]. Further analysis showed P_200_ responses were attenuated under CPS than control condition (*p* = 0.042), and females had significantly larger *S*_1_ P_200_ amplitude than males (*p* = 0.018), but there was no notable difference in *S*_2_ response amplitude between genders (*p* = 0.10).

P_200_ gating ratios and gating difference were evaluated by performing 2 (gender) × 2 (treatment) mixed measure ANOVAs. The results showed no main effects of gender (*p* = 0.26) and treatment (*p* = 0.77) and no interaction (*p* = 0.90) for gating ratios. The data showed similar results for gating differences, but a significant gender effect [*F* (1, 39) = 4.47, *p* = 0.041, *η*2 p = 0.10], with better P_200_ gating function for females.

## 4. Discussion

To characterize the response to the CPS, salivary cortisol and cardiovascular response were assessed. The results revealed significantly higher blood pressures, as well as an increased activity of the hypothalamus-pituitary-adrenal axis (reflected by higher cortisol concentrations) after CPS treatment, compared to the control procedure session. The findings are well in line with the previous studies (e.g., [4, 51]) and indicate the successful induction of a neuroendocrine stress response [1]. Moreover, a comparable basis was demonstrated by the baseline psychological measures across all conditions.

In the paired-click paradigm, sensory gating is operationally defined as the ratio of the amplitude of the response to *S*_2_ divided by that of *S*_1_ or the amplitude difference between the two clicks (e.g., [9–11]). Higher ratios and smaller absolute difference values mean worse sensory gating, which could be a result of lower amplitudes in response to *S*_1_, thus weaker registration functions, or higher amplitudes in response to *S*_2_, thus weaker inhibition with repetition, according to some authors (e.g., [41–43]). In this view, this study showed diminished P_50_ gating due to enhanced amplitudes to *S*_2_ significantly under CPS relative to control condition, while P_50_ amplitudes to *S*_1_ remaining unaffected, which indicated that CPS disrupted subjects' capacity of inhibition with repetition, but not sensory registration. These results were in right accordance with Johnson and Adler [26] and Atchley [30]. In addition, Ermutlu et al. [29] used an oddball paradigm and also found CPS impaired P_50_ sensory gating. As for Woods et al. [11] reporting no evidence on impairment in P_50_ gating, the most likely explanation is that the CPS procedure used in that study was so weak as only 50 seconds of immersing in cold water that the stress stimuli may not be strong enough to trigger the abundant releasing of norepinephrine (NE) and cortisol to disrupt the sensory gating. A much stronger CPS procedure was used in the current study, and both data of blood pressures and saliva samples proved successful induction of a neuroendocrine stress response. As was known, acute stress can lead to the release of NE from widely distributed synapses, including abundant projections to the PFC, and the rapid activation of the prefrontal dopamine system, and on a slightly longer time scale, the release of cortisol, whose receptors are also abundant in the PFC [3, 39, 40, 52]. However, it was proposed that PFC should be the major neural source of P_50_ suppression by many documents (e.g., [9, 38, 53–56]). Thus, it is reasonable that acute stress disrupts P_50_ sensory gating via the adverse effects of stress hormones and neuromodulators on PFC which might result in impairments of the inhibition of redundant information.

However, White and Yee [27] reported a reduction of *S*_1_ P_50_ amplitude during mental arithmetic stressor, which are different from the results of Johnson and Adler [26], Atchley [30] and the current study that acute stress resulted in an increase of *S*_2_ P_50_ amplitude with unchanged *S*_1_ P_50_ amplitude. Given that consciously directing attention toward the clicks enhanced P_50_ amplitude in healthy subjects (e.g., [16, 31]), oral mental arithmetic stressor task during passively listening task makes less attention resources switched to the clicks and might then a reduction of *S*_1_ P_50_ amplitude, while in the cases of Johnson and Adler [26], Atchley [30] and the current study, CPS was used to induce stress state and there was no specific cognitive processes involved.

No gender difference in P_50_ amplitudes and sensory gating was reported in the current study, which was in accordance with previous studies [6, 22,21, 24, 25]. Freedman, Adler, and Waldo [20] also reported no gender difference in P_50_ sensory gating, but they found women had higher P_50_ amplitude to *S*_1_ than men. However, Franks et al. [19] and Hetrick et al. [10] reported women had significantly larger P_50_ responses to *S*_2_, but Fuerst et al. [23] reported men scored higher than women in P_50_ amplitude to *S*_1_. It is not likely to interpret the discrepancy properly by gender difference in the neuroanatomical origins of the auditory P_50_ response and inhibition, because it was documented that there was no known gender difference in brain structures or neuronal systems relevant to P_50_, such as auditory cortex, thalamus, and PFC ([57]; e.g., [9, 10]), and the discrepancy is mostly state dependent. As mentioned earlier, menstrual cycles could affect several cognitive processing. In particular, Goldstein et al. [35, 58] found menstrual cycle modulated women's arousal state with less cortical control over the amygdala during early follicular due to low level of estrogen, but an attenuation of brain activity during midcycle in the presence of higher levels of estrogen. Therefore, given different arousals leading to different baseline P_50_ responses [11, 59] and the interplay between acute stress and sex steroid hormone (for a review, see [60]), the mechanisms under the discrepancy is more likely estrogen level dependent.

The results of the current study showed no gender difference in the CPS effects on P_50_ amplitudes and sensory gating. However, White et al. [12] reported a significant gender difference with a significantly reduced P_50_ to *S*_1_ but not *S*_2_ during mental arithmetic stressor for women, not for men, but they did not report any information about menstrual cycles. Therefore, in addition to the defects in design mentioned earlier, the most likely explanation would be that more early follicular phase women participated in that study, while female subjects used in the current study were all in their midluteal phase (days 16 to 24) during which there was a steady rise in estrogen levels forming a second estrogen peak in menstrual cycles [61] and then women might be more comparable to men in arousal and sensory gating because of more cortical control over the amygdala due to high level of estrogen. Future studies should select healthy female subjects during their different menstrual cycles based on direct measurements of gonadal hormone levels instead of self-reported data to precisely determine menstrual phases to further confirm and extend the present findings.

As reviewed earlier, relatively fewer studies using paired-click procedure focused on N_100_ and P_200_ sensory gating. And the relevant findings were also inconsistent. According to Hetrick et al. [10], women had significantly higher N_100_ amplitudes to *S*_2_ and worse N_100_ sensory gating compared to men, but no significant differences were found at P_200_, and Fuerst et al. [23] found that men had larger N_100_ and P_200_ amplitude to *S*_1_ and better N_100_ and P_200_ sensory gating relative to women. However, other studies reported no gender difference [22, 24]. Data of the current study showed women had larger *S*_1_ N_100_ and P_200_ amplitudes to *S*_1_ and better N_100_ and P_200_ gating function. In addition to the possible effects of arousal due to fluctuations of gonadal hormones mentioned earlier, potential manipulation of attention because of nonstandardized instructions across studies might be an alternative interpretation. Gjini et al. [31] reported attention status had a significant effect on N_100_ and P_200_ amplitudes and gating. However, there is no specific control for attention factors, according to typical instructions in paired-click paradigms. For example, subjects are usually requested to remain awake and try to decrease their eye movements, close (e.g., [10] and the current study) or open eyes, fixate on an object (e.g., [22]) or not, listen passively or intently to clicks (e.g., [22, 24]), keep relaxed or not, and so on. Thus, different baseline attention status due to different emphases in instructions, combined and interacted with fluctuations of gonadal hormones, leads to complex results.

Some studies suggested that in paired-click paradigm N_100_ could reflect initial direction of attention, and P_200_ reflects early allocation of attention, initial conscious experience, and entrance into working memory of the stimulus [31, 62, 63]. Therefore, women in the current study might be more alert, reflected by larger N_100_ amplitudes than men both to *S*_1_ and *S*_2_. And in the end, women allocated more attention resources to relevant information than men, reflected by significantly larger *S*_1_ P_200_ amplitude for females than males. Additionally, the current study also indicated that CPS disrupted allocation of attention resources, reflected by attenuated activation during P_200_ time window for both clicks after CPS than control procedures, which was in accordance with the majority of the findings in attention studies, and should involve the mechanism that acute stress impairs normal selective attention via disruptions of higher PFC functions (as a review, see [64]).

As for the effects of acute stress on sensory gating of N_100_ and P_200_, the current study found no stress effect, which was in accordance with Ermutlu et al. [29]. However, White et al. [12] using mental arithmetic stressor reported disrupted N_100_ gating ratios. In typical paired-click paradigm, only pairs of identical clicks are presented and cognitive processing resources are always sufficient. This might be a possible explanation for no effect of CPS on N_100_ amplitude and gating indices in Ermutlu et al. [29] and this current study, but significant stress effects on N_100_ response and gating ratio in White et al. [12] because of the potential inadequate process resources allocated in paired-click listening task due to concurrent mental arithmetic processing and the impairment of inhibition function due to stressful situation.

At last, the data showed significantly longer latencies to *S*_1_ than *S*_2_ both for N_100_ and P_200_ and a similar trend for P_50_ although not statistically significant in this current study, which were in accordance with former studies [10, 23, 24, 65]. We cannot interpret the results properly due to scarcity of relevant documents. Probably, peak latencies reflect the depth of information processing and cognitive process to *S*_2_ is incomplete because of sensory gating, and as a result, the latency to *S*_1_ is longer than *S*_2_. The data of this study also showed a trend of longer N_100_ latencies for men than women, and it might be the fact that the depth of cognitive process during N_100_ time window was less for women than men, although as mentioned earlier, women might be more alert and allocate more attention resources to relevant information than men, according to larger N_100_ and P_200_ amplitudes than men both to *S*_1_ and *S*_2_. In a word, peak latencies in the paired-click paradigm need more concerns and relevant data need to be better understood in the future, but at least the current latency data are in accordance with former studies, which assures in some extent that the components of P_50_, N_100_, and P_200_ are extracted precisely.

As for the application of paired-click paradigm in neural pathological studies, based on this study, more emphasis should be paid on the control of all kinds of factors that might induce subjects a stressful state, and we suggest the importance of monitoring stress and anxiety levels of subjects, and furthermore, N_100_ and P_200_ gating indices should be cautiously used because of their strong correlations with high-order cognitive process. But no matter how does one think of the applications of the P_50_, N_100_, and P_200_ sensory gating in pathological fields, the primary focus of the work is to standardize the recording of gating data and the extracting procedure of the components.

## Figures and Tables

**Table 1:** Summary of studies investigating auditory sensory gating concerning gender difference and acute stress effects.

Study	Subjects	Stress and recording timing	Components	Findings
Franks et al. [19]	22f, 21m	No acute stress	P_50_	Women showed less suppression of the P_50_ than men in a mixed sample of manic and normal subjects
Freedman et al. [8, 20]	73f, 90m	No acute stress	P_50_	Women had a higher P_50_ amplitude to *S*_1_ than men across all age groups, without gender differences in P_50_ ratios
Hetrick et al. [10]	30f, 30m	No acute stress	P_50_, N_100_, P_200_	Women had higher P_50_ and N_100_ amplitudes to *S*_2_ and worse gating for P_50_ and N_100_ without gender effects at P_200_
Rasco et al. [21]	25f, 25m	No acute stress	P_50_	No gender differences in P_50_ amplitudes, latencies, and sensory gating across all age groups
Clementz et al. [22]	15f, 25m	No acute stress	P_50_, N_100_	No gender differences in P_50_ and N_100_ amplitudes, latencies, and sensory gating
Brinkman et al. [6]	67f, 45m	No acute stress	P_50_	No gender differences of P_50_ latency, amplitude, and inhibition across all age groups
Fuerst et al. [23]	38f, 29m	No acute stress	P_50_, N_100_, P_200_	Men scored higher than women in P_50_, N_100_, or P_200_ amplitude to *S*_1_ and in all gating differences
Lijffijt et al. [24]	34f, 26m	No acute stress	P_50_, N_100_, P_200_	No gender effects in P_50_, N_100_, or P_200_ amplitudes and sensory gating
Thomas et al. [25]	21f, 13m	No acute stress	P_50_	No gender effects in P_50_ amplitudes and P_50_ gating ratios
Johnson and Adler [26]	2f, 8m	Immediately after 2 min CPS	P_50_	*S*_2_ P_50_ amplitude increased, and the gating ratio also increased, after exposure to CPS
White et al. [27]	7f, 6m	During mental arithmetic stressor	P_50_	P_50_ gating was reduced, due to a reduction of *S*_1_ P_50_ amplitude, relative to nonstress task
Yee et al. [28]	9f, 11m	During mental arithmetic stressor	P_50_	Stressor disrupted P50 sensory gating without differential effects on P_50_ amplitudes to *S*_1_ and *S*_2_
^∗^Ermutlu et al. [29]	7f, 8m	During 5 min CPS	P_50_, N_100_	CPS impaired P_50_ sensory gating without effect on N_100_ gating (deviant/standard)
Woods et al. [11]	21f, 9m	Immediately after 50 s CPS	P_50_	*S*_1_ P_50_ amplitude decreased, without effect on P_50_ gating ratio, after exposure to CPS
Atchley [30]	20f, 10m	Immediately after 2 min CPS	P_50_	P_50_ gating ratios increased after CPS
White et al. [12]	13f, 16m	During mental arithmetic stressor	P_50_, N_100_	*S*_1_ P_50_ was reduced for women, whereas men showed reductions for both clicks, and women showed disrupted P_50_ N_100_ gating whereas men only disrupted N_100_ gating, during stress compared to baseline

^∗^Using oddball paradigm; f: female; m: male; CPS: cold pressor stress.

**Table 2:** Preexperiment mood and physiological measurements before and after control or CPS treatment.

	Gender	Positive affect	Negative affect	State anxiety	Depression	Baseline HR	HR after 4 min
CON (*M*, SD)	Male	29.3 (8.3)	16.1 (5.1)	35.9 (8.4)	6.9 (6.2)	69 (13)	66 (16)
	Female	28.9 (8.0)	18.6 (5.3)	35.2 (8.8)	7.9 (6.5)	75 (8)	73 (11)
CPS (*M*, SD)	Male	30.0 (6.8)	16.7 (4.6)	36.0 (7.4)	7.3 (5.4)	62 (11)	61 (9)
	Female	29.6 (7.8)	17.3 (5.4)	33.0 (9.4)	8.1 (4.9)	70 (9)	67 (10)

	Gender	Baseline DBP	DBP after 4 min	Baseline SBP	SBP after 4 min	Baseline CORT	CORT after 15 min
CON (*M*, SD)	Male	63 (6)	63 (5)	112 (9)	111 (9)	5.0 (1.6)	5.0 (1.5)
	Female	62 (6)	61 (4)	101 (10)	100 (7)	6.2 (2.1)	6.1 (2.0)
CPS (*M*, SD)	Male	67 (6)	69 (7)	115 (7)	116 (7)	5.8 (1.5)	6.1 (1.7)
	Female	62 (5)	66 (5)	101 (7)	105 (7)	6.6 (1.8)	7.3 (2.3)

Values represent means (*M*) and standard deviations (SD); CON: control condition; CPS: cold pressor stress; HR: heart rate (beats per minute); DBP: diastolic blood pressure (mmHg); SBP: systolic blood pressure (mmHg); CORT: cortisol (nmol/L).

**Table 3:** Amplitudes, latencies, gating ratios, and differences for P_50_, N_100_, and P_200_, *M* (SD).

		Treatment	*S*_1_		*S*_2_		Gating ratio (*S*_2_/*S*_1_ × 100)	Gating difference
			Latency	Amplitude	Latency	Amplitude		
P_50_	Male	CON	76 (9)	4.1 (2.6)	74 (10)	2.0 (2.0)	52 (40)	2.1 (2.4)
		CPS	75 (8)	3.6 (3.3)	74 (9)	3.1 (4.0)	94 (85)	0.5 (1.4)
	Female	CON	73 (7)	5.5 (4.0)	71 (11)	3.2 (3.1)	59 (28)	2.3 (2.6)
		CPS	72 (7)	4.9 (3.9)	72 (10)	4.2 (3.6)	95 (62)	0.6 (1.9)
N_100_	Male	CON	122 (12)	–7.7 (4.8)	117 (16)	–1.9 (2.6)	39 (26)	5.8 (3.6)
		CPS	121 (14)	–7.7 (4.7)	118 (18)	–1.9 (2.5)	43 (37)	5.8 (3.6)
	Female	CON	118 (11)	–12.7 (11.6)	111 (14)	–3.8 (4.4)	37 (32)	8.9 (8.8)
		CPS	116 (12)	–12.9 (9.7)	110 (13)	–3.5 (4.1)	34 (20)	9.4 (7.3)
P_200_	Male	CON	196 (17)	8.8 (7.6)	188 (19)	5.1 (2.9)	67 (49)	3.7 (6.5)
		CPS	198 (18)	7.0 (4.4)	191 (21)	4.5 (2.3)	70 (54)	2.5 (4.3)
	Female	CON	193 (20)	12.9 (7.0)	191 (26)	6.6 (3.0)	54 (24)	6.3 (5.3)
		CPS	198 (15)	11.7 (5.7)	189 (18)	5.2 (2.0)	56 (37)	6.4 (6.1)

## Data Availability

All data included in this study are available upon request by contact with the corresponding author.
